# Dr. Burnham’s Report of the above Operation

**Published:** 1867-12

**Authors:** 


### DR. BURNHAM'S REPORT OF THE OPERATION.

Mrs. H., of Chicago, aged —, has had a tumor in the lower part of the abdomen for several years, during the last three or four of which it has been complicated with ascites, to such an extent that she has been "tapped" several times to relieve her from the pressure of the accumulated fluid; the last operation having taken place about a week prior to my seeing her. At each time about twenty pounds of serous fluid had been removed, leaving a hard, irregular, but movable tumor, about the size of a fœtal head at full term, occupying the lower part of the abdomen and pelvis. This had increased very slowly, but seemed to have become more irregular and nodulated after each tapping.

She had been treated by several of the profession in Chicago, and had, from time to time, been seen in consultation by the best surgical and medical talent in the city; and, although the symptoms were very obscure, and the appearances varied at the different examinations which were made, yet the general opinion was that she had a multilocular ovarian tumor. She was very feeble and greatly emaciated, but was still able, during a great portion of the time, to go about her house.

I first saw her on the 29th day of May last, by the invitation of Dr. D. Dodge (who had, at a previous period, been her attending physician, although not at that time in charge), and after examination and learning the history of the case, I advised an exploring operation by a short incision through the abdominal walls, with a view to ascertain the kind, character, and extent of the adhesions, if any there were, so that, if owing to them, it was not deemed feasible to attempt the removal of the tumor, the patient might be subjected to the least possible risk. On the following day, I met Dr. Dodge, the two Drs. Clark, and Dr. Seeley in consultation, and they fully concurred with my views concerning an operation. Accordingly, the patient was placed on a lounge, and chloroform was administered until she was fully under its influence. I then made an incision through the integuments down to the peritoneum, extending from an inch below the umbilicus three inches downward in the median line, then, raising the peritoneum carefully, I divided it and passed the canula of a No. 10 trocar into the abdominal cavity, removing about fifteen pounds of a serous fluid, after which I increased the opening in the membrane, so that it corresponded with the original incision, by which means I was enabled to gain a free examination of the tumor, which I found sustained by a pedicle from the broad ligament of the uterus. There were, also, firm adhesions to the intestines to a considerable extent, and one of the Fallopian tubes was attached throughout its entire length. I thought, however, that these might be separated, and, with that view, extended my first incision three inches upward (avoiding the umbilicus), and about two inches downward, thus gaining ample room to turn the mass around, and, by presenting its smallest diameter, to bring it to the surface, that I might the better remove the adhesions. I then carefully dissected off the attachments to the intestines and the Fallopian tube, being obliged to ligate and remove a portion of the omentum, which was so thoroughly incorporated with the tumor that it could not be separated. I next passed a double silk ligature around the pedicle and drew it tightly, holding it firmly for some time before securing it, to prevent the duplication of membrane from slipping. I then tied it and separated the pedicle about a-half inch from the ligature.

The wound was dressed by approximating the edges with five (5) wire sutures (bringing the ligature out at the bottom), and covered with straps of adhesive plaster, extending from one side to the other; above these, a compress of cotton batting was applied, and the whole secured by a broad bandage. The patient was then removed to bed and a mild anodyne administered, together with nutritious drinks, *p. r. n.* She was left in charge of Dr. Seeley.

In conclusion, I wish to return my warmest thanks for the very kind and efficient aid rendered me by the medical gentlemen above enumerated, during this difficult and somewhat tedious operation.

